# Gonococcal cervicitis among women seeking fertility care in Uganda: Prevalence, antimicrobial susceptibility patterns, and associated factors in a multicenter cross-sectional study

**DOI:** 10.1371/journal.pgph.0006796

**Published:** 2026-09-08

**Authors:** Mohamed Mire Amina, Musa Kasujja, Marie Pascaline Sabine Ishimwe, Amina Aden Ahmed, Hudaifa Iman Yusuf, Theodore Nteziyaremye, Bahja Ahmed Mumin, Ali Abdow Hafsa, Mohamed Farah Ismail, Adam Abdrahim Sulaiman Ebaid, Jean de Dieu Rukamba, Ronard Musinguzi, Sawda Abdikarim Sheikh Isse, Theoneste Hakizimana

**Affiliations:** 1 Department of Obstetrics and Gynecology, Kampala International University, Ishaka, Uganda; 2 Department of Pediatrics and Child Health, Kampala International University, Ishaka, Uganda; 3 Department of Ophthalmology, Kampala International University, Ishaka, Uganda; 4 Department of Sciences, University of Rwanda, Kigali, Rwanda; Clínica de Familia La Romana, DOMINICAN REPUBLIC

## Abstract

Gonococcal cervicitis can contribute to reproductive tract complications and infertility, yet evidence from fertility-care settings in Uganda remains limited. This study determined its prevalence, antimicrobial susceptibility patterns, and associated factors among women seeking fertility care at three tertiary hospitals in Uganda. We conducted a multicenter cross-sectional study involving 402 women seeking fertility care at three tertiary hospitals in Uganda from 10th September 2025 and 10th January 2026. Endocervical swabs were cultured for *Neisseria gonorrhoeae*, and isolates underwent antimicrobial susceptibility testing. Factors associated with gonococcal cervicitis were assessed using bivariable and multivariable logistic regression. Of the 402 participants, 34 had culture-confirmed gonococcal cervicitis, for an overall prevalence of 8.5% (95% CI, 6.1%–11.6%). Site-specific prevalence was 10.4% at the Jinja Regional Referral Hospital and 7.5% at both Lira and Kayunga Regional Referral Hospitals, with no significant between-site difference (p = 0.598). Among the 34 isolates, susceptibility was highest to ceftriaxone and azithromycin (94.1% each), whereas resistance was common to penicillin (64.7%), ciprofloxacin (58.8%), and tetracycline (58.8%). Independent factors associated with gonococcal cervicitis were having three or more lifetime sexual partners (adjusted odds ratio [AOR], 5.25; 95% CI, 1.65–16.72), vaginal douching (AOR, 5.59; 95% CI, 1.83–17.03), current oral contraceptive use (AOR, 8.32; 95% CI, 2.94–23.54), HIV seropositivity (AOR, 5.00; 95% CI, 1.82–13.76), and a new sexual partner in the previous 3 months (AOR, 7.92; 95% CI, 2.68–23.41). Gonococcal cervicitis was common among women seeking fertility care at these Ugandan tertiary hospitals. The observed resistance to penicillin, ciprofloxacin, and tetracycline, together with preserved susceptibility to ceftriaxone and azithromycin, supports culture-informed treatment and targeted screening in fertility clinics.

## Introduction

Infertility remains a major reproductive health problem worldwide and disproportionately affects women in low-resource settings, where underlying causes are often identified late and access to advanced fertility services remains limited [1]. Among infectious contributors, *Neisseria gonorrhoeae* is particularly important because cervical infection is frequently asymptomatic but can still ascend to the upper genital tract. Gonococcal infection can therefore lead to endometritis, pelvic inflammatory disease (PID), tubal scarring, ectopic pregnancy, chronic pelvic pain, infertility, and adverse pregnancy outcomes, including subclinical reproductive damage that may only become apparent during infertility evaluation [2–5]

Infertility is also an important reproductive health concern in Uganda. Analysis of three nationally representative Uganda Demographic and Health Surveys estimated an overall infertility prevalence of 6.4%, while secondary infertility remained relatively unchanged at approximately 7% between 2006 and 2016 [6]. Infection-related tubal damage following untreated sexually transmitted infections and pelvic inflammatory disease, complications of post-abortion and postpartum infections, ovulatory disorders, and male-factor infertility are among the major contributors to infertility in Uganda and sub-Saharan Africa [7,8]. Uganda’s national reproductive health guidelines similarly recognize sexually transmitted infections, inadequately managed post-abortion infections, and puerperal sepsis as important preventable causes of infertility [8]. Beyond its biological consequences, infertility imposes substantial psychosocial and economic burdens. Recent qualitative evidence from Uganda describes stigma, emotional distress, prolonged help-seeking, delayed diagnosis, and challenges navigating fertility services among affected individuals and couples [9]. Although basic infertility evaluation and treatment are increasingly available in Ugandan hospitals, access to comprehensive diagnostic investigations and advanced fertility care remains limited. A nationwide survey found that hormonal testing, hysterosalpingography, semen analysis, reproductive surgery, intrauterine insemination, and assisted reproductive technologies were substantially less available in public hospitals than in private-for-profit facilities. Advanced infertility services were available in only 6.3% of surveyed hospitals and were concentrated mainly in urban referral centers and the Central region [10]. Consequently, limited service availability together with high out-of-pocket costs create important financial and geographical barriers that may delay diagnosis, treatment, and referral for specialized fertility care [9,10]

This biological pathway is particularly relevant in fertility care because a clinical history alone may underestimate prior infectious injury. Many women with tubal-factor infertility do not report previous PID, supporting the view that missed or minimally symptomatic lower genital tract infections can still produce important upper-tract sequelae [2]. Consistent with this, a global systematic review of infertile populations estimated a pooled mean prevalence of current gonorrhea of 2.2% worldwide and 5.0% in Africa, with a higher prevalence among women with tubal-factor infertility and those with secondary infertility [11]. Cross-sectional studies outside of Africa have also supported this reproductive link: gonorrhea prevalence was 16.9% among infertile women in one Iranian hospital-based study and was associated with infertility and ectopic pregnancy in another Tehran cohort of women with genitourinary infection and pregnancy-related complications [12,13].

The setting in which women are sampled strongly shapes the observed prevalence, and this distinction is critical when interpreting fertility-clinic data. In broad community or preconception populations, gonococcal prevalence may be very low, such as 0.06% among women preparing to conceive in Chongqing and 0.17% in a population-based study of women in Shenzhen, China [14,15]. In contrast, prevalence is substantially higher in symptomatic sexually transmitted infection populations and in clinic-based reproductive health settings. In East Africa and similar resource-limited settings, reported prevalences include 23.6% among symptomatic outpatients in northern Uganda, 10.0% in Mekelle, Ethiopia, 9.8% in Jimma, Ethiopia, 6.3% among symptomatic female sex workers in Nairobi, and 4.9% among symptomatic patients in Lyatonde, Uganda [16–20]. These differences are not merely geographic; they reflect differences in symptom enrichment, background risk, and diagnostic strategy. Fertility clinics may therefore represent a distinct epidemiologic niche in which previously undetected infection, cumulative reproductive damage, and active cervical infection intersect.

Gonococcal infection in women is also clinically heterogeneous. The presentation ranges from asymptomatic carriage to overt cervicitis, and this variation may be shaped in part by the cervicovaginal microbial environment. In women with gonococcal infection, asymptomatic presentation has been reported more often in those with Lactobacillus-dominant communities, whereas symptomatic infection has been associated more often with diverse bacterial vaginosis-like communities enriched with taxa such as *Prevotella*, *Sneathia*, *Mycoplasma hominis*, and BV-associated bacterium [21]. Related work in African cohorts has shown that Lactobacillus-depleted cervicovaginal states are associated with poorer reproductive outcomes and greater vulnerability to sexually transmitted infection, providing a plausible ecological context for why some cervical infections may be more inflammatory, more persistent, or more clinically apparent than others [22,23].

The sub-Saharan African context further strengthens the case for laboratory-based diagnosis. Across the region, sexually transmitted infection care still relies heavily on syndromic management, yet many gonococcal infections in women are asymptomatic. This creates a dual problem: women without gonorrhea may still be exposed to antibiotics, whereas women with true but minimally symptomatic infection may remain untreated. The result is continued transmission, persistent reproductive tract inflammation, and avoidable antimicrobial pressure that may accelerate resistance [4,24,25].

In Uganda, STI prevention and care are integrated within general outpatient and sexual and reproductive-health services, including HIV, antenatal, and family-planning clinics [8]. National guidelines recommend syndromic STI management, particularly where organism-specific diagnostic testing is unavailable [26]. Although this approach enables treatment in facilities with limited laboratory capacity, it may miss cervical gonococcal infections because many infections in women are asymptomatic or cause only mild, nonspecific symptoms [4]. Consequently, reliance on symptoms alone may leave some women undiagnosed [4]. Given the documented burden of gonorrhoea among infertile populations, particularly in Africa and among women with tubal-factor, mixed or unexplained infertility [11], fertility clinics may provide an important opportunity for targeted laboratory-based STI assessment.

Antimicrobial resistance provides a second major reason to study gonococcal cervicitis in fertility care. *N. gonorrhoeae* has progressively acquired resistance to nearly every antibiotic class used against it, making effective treatment increasingly dependent on surveillance and stewardship [27–30]. Global surveillance has shown recurring resistance patterns across regions, along with geographically distinct emerging threats, underscoring that resistance trajectories cannot be assumed to be uniform across settings [31]. In sub-Saharan Africa, a systematic review has indicated very high resistance to ciprofloxacin, tetracycline, and penicillin, with azithromycin resistance also reported and reduced cephalosporin susceptibility emerging in some settings [24]. Uganda reflects this broader pattern. Mabonga et al. [32] documented complete ciprofloxacin resistance among viable Kampala isolates and preserved ceftriaxone and cefixime susceptibility by agar dilution, while earlier Ugandan data from Lacor showed substantial loss of susceptibility to ampicillin, tetracycline, erythromycin, and ciprofloxacin [17,32]. These trends are clinically important in fertility settings, where ineffective treatment may allow ongoing cervical infection and further reproductive tract injury.

Beyond its clinical consequences, gonorrhea remains an important public health challenge because of its contribution to ongoing transmission, antimicrobial resistance, and reproductive morbidity. The WHO estimates that millions of new gonococcal infections occur annually worldwide, with the highest burden occurring in low- and middle-income countries where access to laboratory diagnosis remains limited. Untreated infection can lead to pelvic inflammatory disease, tubal factor infertility, chronic pelvic pain, and adverse reproductive outcomes, while increasing antimicrobial resistance threatens the effectiveness of currently recommended treatments. Strengthening surveillance of gonococcal infection and resistance patterns is therefore considered a key component of global sexually transmitted infection control strategies [33,34].

Both N. gonorrhoeae and C. trachomatis may be asymptomatic in women and can contribute to pelvic inflammatory disease, ectopic pregnancy, and infertility. This study focused specifically on N. gonorrhoeae because the growing threat of antimicrobial resistance required culture-based isolation and phenotypic susceptibility testing, for which evidence from Ugandan fertility clinics was lacking. Testing for C. trachomatis was not undertaken because nucleic acid amplification testing was not available within the study resources. Consequently, this study does not describe the complete burden of sexually transmitted infections in this population.

Important gaps remain regarding the burden, susceptibility profile, and clinical correlates of culture-confirmed GC cervicitis among women seeking fertility care in Uganda. We conducted a multicenter cross-sectional study to determine the prevalence of GC cervicitis, assess antimicrobial susceptibility patterns of *N. gonorrhoeae* isolates, and identify factors associated with infection among women seeking fertility care at tertiary hospitals in Uganda.

## Materials and methods

### Ethics statement

Ethical approval for this study was obtained from the Kampala International University Research Ethics Committee (KIU-REC) under approval number KIU-REC-2025–1732. Administrative permission to conduct the study was granted by Jinja Regional Referral Hospital, Lira Regional Referral Hospital, and Kayunga Regional Referral Hospital. The study was also registered with the Uganda National Council for Science and Technology (UNCST).

Written informed consent was obtained from all participants before enrolment after providing detailed information about the study in a language they understood. Participation was entirely voluntary, and all participants were aged 18 years or older and provided consent personally. Confidentiality was maintained through the use of unique study identification numbers and secure storage of questionnaires, laboratory records, and other study-related documents. The study was conducted in accordance with the ethical principles outlined in the Declaration of Helsinki. Participants with positive cultures were counselled and referred for treatment with cefixime 400 mg orally as a single dose or ceftriaxone 1 g intravenously as a single dose, plus doxycycline 100 mg twice daily for seven days and metronidazole 2 g orally as a single dose. Erythromycin replaced doxycycline during pregnancy. Partner evaluation and treatment were encouraged to prevent reinfection

### Study design and setting

We conducted a multicenter, hospital-based cross-sectional study at Jinja, Lira, and Kayunga Regional Referral Hospitals between 10^th^ September 2025 and 10^th^ January 2026. These public tertiary hospitals serve different geographical regions of Uganda. Jinja Regional Referral Hospital is a 500-bed facility serving approximately 4.6 million people in Jinja City and 11 surrounding districts. Lira Regional Referral Hospital has 401 beds and serves approximately 2.5 million people across nine districts and Lira City. Kayunga Regional Referral Hospital serves more than 7 million people across six districts in central Uganda.

All three hospitals receive referrals from district hospitals and lower-level health facilities and provide specialist gynecology and fertility services through outpatient clinics. Their microbiology laboratories can perform bacterial culture, organism identification, and routine antimicrobial susceptibility testing. However, long travel distances, transport costs, hospital congestion, and out-of-pocket costs for investigations may limit access for some women. The hospitals were purposively selected because they had established fertility services, functional microbiology laboratories, and served populations from different regions of Uganda, thereby broadening the geographical relevance of the study findings. Jinja, Kayunga and Lira Regional Referral Hospitals are located in eastern, central and northern Uganda, respectively. Based on their geographical coordinates, the hospitals are separated by approximately 46–205 km in straight-line distance and serve geographically and socially diverse urban and rural referral populations.

### Study period and participants

Data were collected from September 10, 2025, to January 10, 2026. Eligible participants were women presenting for the evaluation of primary or secondary infertility after at least 12 months of regular unprotected sexual intercourse. Women were included if they met the infertility definition and provided written informed consent. The exclusion criteria were refusal to participate, menstruation at the time of sampling, prior hysterectomy, inability to undergo speculum examination, and incomplete study data. Women reporting oral, injectable or intravaginal/topical antibiotic use during the preceding two weeks were also excluded because recent antimicrobial exposure could suppress gonococcal growth and reduce culture sensitivity.

### Sample size and sampling

The sample size was estimated using the single population proportion formula described by Kish and Leslie (1965): n = Z² p (1 − p)/d², where n is the required sample size, Z is the standard normal deviate at a 95% confidence level (1.96), p is the estimated prevalence, and d is the margin of error, set at 0.05. Using an estimated Neisseria gonorrhoeae prevalence of 8.4% reported in Tanzania [35], the initial sample size was calculated as follows:


$$\begin{array}{c} \text{n}=({\text{1}.\text{96}}^{\text{2}}\times \text{0}.\text{084}\times (\text{1}\;-\;\text{0}.\text{084}))/{\text{0}.\text{05}}^{\text{2}}=\text{119}. \end{array}$$

After allowing 10% for nonresponse, the minimum target was approximately 130 participants. This calculation did not incorporate a design effect because a reliable estimate of within-hospital correlation was unavailable during planning. Recruitment continued throughout the prespecified study period, resulting in a final sample of 402 women, with 134 recruited from each hospital.

On each recruitment day, potentially eligible women were identified sequentially from the fertility or gynecology clinic attendance register. Following selection of a random starting position from the first three eligible attendees, every third eligible woman was approached by a trained research assistant in a private area after routine clinic registration. Eligibility was confirmed, and the study was explained in the participant’s preferred language. Written informed consent was obtained before administration of the questionnaire or collection of an endocervical specimen. Women who declined participation continued to receive routine clinical care without disadvantage.

### Data collection procedures

After providing written informed consent, participants were interviewed by trained bilingual research assistants using a structured, interviewer-administered questionnaire. The questionnaire collected sociodemographic, sexual and behavioural, reproductive, and relevant clinical information. Because no single standardized instrument addressed all the study objectives, the questionnaire was developed from the study objectives and informed by previous studies on gonococcal infection, infertility, and related reproductive-health factors. It was reviewed for relevance and clarity and pilot-tested before the main study. Findings from the pilot test were used to refine the wording, response options, and sequence of questions.

The questionnaire was administered in English or the participant’s preferred local language. Interviews were conducted in Lusoga at Jinja, Luganda at Kayunga, and Lango at Lira by research assistants fluent in the respective languages. All research assistants received standardized training in participant recruitment, informed consent, interviewing, questionnaire completion, confidentiality, and accurate recording of responses. The same questionnaire and interview procedures were used across the three hospitals to reduce interviewer-related variation.

Consistent eligibility criteria and systematic recruitment were used to reduce selection bias. Because sensitive behavioural and reproductive information was self-reported, recall and social-desirability bias remained possible. Interviews were therefore conducted privately using neutral, non-judgmental language. Standardized procedures for specimen collection, transportation, culture, identification, and antimicrobial susceptibility testing were applied across the study sites to reduce laboratory variation and potential misclassification. Before the main study, the questionnaire was pilot-tested among 40 women, representing approximately 10% of the planned sample

### Outcome and covariates

Infertility was defined according to the World Health Organization as failure to achieve pregnancy after at least 12 months of regular unprotected sexual intercourse. Time since infertility diagnosis was considered a separate variable and was defined as the participant-reported interval between clinical confirmation of infertility and enrolment in the study. It was categorized as <1 year, 1–3 years, and >3 years to distinguish recently diagnosed, intermediate, and longer-standing infertility. Therefore, the < 1-year category referred to time since clinical diagnosis and not to the duration of attempts to conceive.

The primary outcome was gonococcal cervicitis, defined as the isolation of Neisseria gonorrhoeae from an endocervical swab using culture and standard microbiological identification procedures. Prespecified explanatory variables included sociodemographic characteristics (age, marital status, educational level, occupation, and monthly household income); sexual and behavioural characteristics (alcohol use, age at first sexual intercourse, lifetime number of sexual partners, a new sexual partner during the preceding three months, and vaginal douching during the preceding three months); obstetric and gynaecological characteristics (abnormal vaginal discharge during the preceding three months, previous ectopic pregnancy, oral contraceptive use, and time since infertility diagnosis); and clinical characteristics (HIV status as per patient records, history of anaemia, and body mass index [BMI]).

These variables were selected before analysis based on published epidemiological evidence, biological plausibility, and their relevance to gonococcal exposure or reproductive morbidity. Sexual-partnership variables were included as measures of potential exposure, while HIV status was considered because HIV and gonococcal infection may occur within overlapping sexual networks. Vaginal douching was included because of its potential effect on the cervicovaginal environment. Previous ectopic pregnancy and time since infertility diagnosis were included to reflect the participant’s reproductive history, while abnormal vaginal discharge was included as a possible clinical presentation of cervical infection, recognizing that gonococcal infection in women may also be asymptomatic.

Age was categorized as <20, 20–29, 30–39, and ≥40 years to provide clinically meaningful reproductive-age groups while retaining sufficient observations for analysis. Formal employment referred to salaried work within a government institution, registered private organization, or another formally constituted employer. Informal employment included small-scale trading, subsistence farming, casual labour, domestic work, and self-employment without a formal contract. Participants without a current income-generating occupation were categorized as unemployed. Monthly household income was grouped using prespecified thresholds of 55USD and 110USD.

Weight was measured using a calibrated weighing scale, and height was measured using a stadiometer. BMI was calculated as weight in kilograms divided by height in meters squared and categorized according to WHO criteria as underweight (<18.5 kg/m²), normal weight (18.5–24.9 kg/m²), overweight (25.0–29.9 kg/m²), or obesity (≥30.0 kg/m²).

Vaginal douching was defined as the intentional introduction of water, another liquid, or a cleansing product into the vagina for cleansing or perceived hygiene at least once during the three months preceding enrolment. It was recorded as a binary variable; information on frequency, products used, volume, and method of application was not collected. Alcohol use was defined as the consumption of any alcoholic beverage during the preceding three months and was also recorded as a binary variable. The questionnaire did not distinguish occasional, weekly, daily, or heavy alcohol consumption.

### Specimen collection and laboratory methods

Endocervical swabs were collected by trained clinicians using sterile specula and swabs under aseptic conditions. Each specimen was labelled with a unique study identification number and promptly transported to the onsite microbiology laboratory, where it was inoculated onto selective Modified Thayer–Martin agar. Culture plates were incubated at 35°C–37°C in approximately 5% carbon dioxide for 24–48 hours. The available study records did not document the transport medium, transport temperature, exact collection-to-inoculation interval, specimen-rejection threshold or use of a cold chain; therefore, these details could not be reconstructed retrospectively. Isolates were identified as Neisseria gonorrhoeae based on characteristic colony morphology, Gram-negative diplococci on Gram staining and a positive oxidase reaction.

Ceftriaxone was included because extended-spectrum cephalosporins remain central to effective gonorrhoea treatment. Azithromycin was included because of its historical role in combination therapy and the importance of monitoring emerging macrolide non-susceptibility. Ciprofloxacin, penicillin, and tetracycline were included to characterize resistance to previously use antimicrobial classes and to allow comparison with earlier Ugandan, regional, and global surveillance findings. Antibiotic selection was also guided by disc availability and the testing capacity of the participating laboratories.

Antimicrobial susceptibility testing was performed using the Kirby–Bauer disk-diffusion method on GC agar base supplemented with 1% defined growth supplement. A direct colony suspension equivalent to a 0.5 McFarland turbidity standard was uniformly inoculated onto the agar surface. Commercial antimicrobial discs containing ceftriaxone 30 µg, azithromycin 15 µg, ciprofloxacin 5 µg, tetracycline 30 µg, and penicillin 10 units were applied. Plates were incubated at 35°C–37°C in approximately 5% carbon dioxide for 20–24 hours, after which inhibition-zone diameters were measured in millimeters. GC agar base supplemented with 1% defined growth supplement is the standardized medium for gonococcal susceptibility testing. WHO laboratory guidance

Zone diameters were interpreted according to CLSI M100, 35th edition (2025). The susceptible, intermediate, and resistant thresholds were ≥47, 27–46, and ≤26 mm for penicillin; ≥ 41, 28–40, and ≤27 mm for ciprofloxacin; and ≥38, 31–37, and ≤30 mm for tetracycline, respectively. CLSI provides susceptible-only disk-diffusion thresholds of ≥35 mm for ceftriaxone and ≥30 mm for azithromycin. Isolates with zone diameters below these thresholds were therefore reported as below the susceptible-only threshold and were not classified as intermediate or resistant because MIC confirmation was not performed.

Neisseria gonorrhoeae ATCC 49226 was used for internal quality control. The applicable CLSI quality-control zone ranges were 39–51 mm for ceftriaxone, 30–38 mm for azithromycin, 48–58 mm for ciprofloxacin, 30–42 mm for tetracycline, and 26–34 mm for penicillin. Individual quality-control zone measurements and documentation of repeated testing runs were not available in the study dataset; therefore, the occurrence or absence of quality-control discordance could not be determined retrospectively. CLSI M100

Participants with positive gonococcal cultures were counselled and treated according to the Ugandan national STI treatment guidelines in force during the study period. Partner notification and treatment were encouraged to reduce the risk of reinfection. Because follow-up was outside the cross-sectional study procedures, routine microbiological test-of-cure was not undertaken. Culture was selected because viable isolates were required for phenotypic antimicrobial susceptibility testing. Nevertheless, culture is generally less sensitive than nucleic acid amplification testing, particularly when bacterial load is low or specimen transportation and processing conditions are suboptimal.

### Quality control

Quality-control measures were applied throughout participant recruitment, questionnaire administration, specimen handling, laboratory testing, and data management. Before data collection, research assistants and laboratory personnel from the three study hospitals received standardized training on the study procedures. The same study definitions, questionnaire, specimen-collection procedures, and harmonized laboratory protocols were used across all three sites. Endocervical specimens were labelled with unique study identification numbers and promptly transported to the respective hospital laboratories under standardized conditions. Neisseria gonorrhoeae ATCC 49226 was used as the internal quality-control strain for antimicrobial susceptibility testing. Quality-control zone diameters were assessed against the CLSI acceptable ranges of 39–51 mm for ceftriaxone, 30–38 mm for azithromycin, 48–58 mm for ciprofloxacin, 30–42 mm for tetracycline, and 26–34 mm for penicillin. However, individual quality-control zone measurements and documentation of repeated testing runs were unavailable for retrospective review. Therefore, it was not possible to determine whether any out-of-range quality-control result or repeat testing occurred. Completed questionnaires were reviewed daily by the principal investigator for completeness and internal consistency. Data-entry records were cross-checked against the completed questionnaires, and regular supervision was conducted throughout the study to assess adherence to the approved protocol.

### Data management and statistical analysis

Data were entered, cleaned, and analyzed using Stata version 17. Descriptive statistics were used to summarize participant characteristics. Prevalence of gonococcal cervicitis was calculated as the proportion of culture-confirmed cases among all tested participants. Participants with incomplete study data were excluded before analysis; accordingly, the final analytic dataset comprised 402 women with complete questionnaire and laboratory data. Bivariable logistic regression was used to examine the associations between explanatory variables and GC. Variables with *p* < .20 were entered into a multivariable logistic regression model. Adjusted odds ratios with 95% confidence intervals were reported. Multicollinearity was assessed using variance inflation factors based on the same indicator-variable coding used in the multivariable model. A VIF greater than 5 was considered potentially problematic. The maximum individual VIF was 3.20, the minimum tolerance was 0.313, and the maximum adjusted generalized VIF was 1.17. Pairwise associations among categorical predictors were weak, with a maximum Cramér’s V of 0.155. Therefore, no variable was removed because of multicollinearity. No sensitivity analyses were performed. Two prespecified multiplicative interactions were evaluated: HIV status × new sexual partner in the preceding three months and vaginal douching × abnormal vaginal discharge in the preceding three months. Each interaction was assessed separately by comparing nested main-effects and interaction models using a one-degree-of-freedom likelihood-ratio test. Both constituent main effects were retained during each interaction test. Because only 34 culture-positive outcomes were observed, the interaction analyses were considered exploratory.

## Results

### Participant characteristics

**Fig 1:**
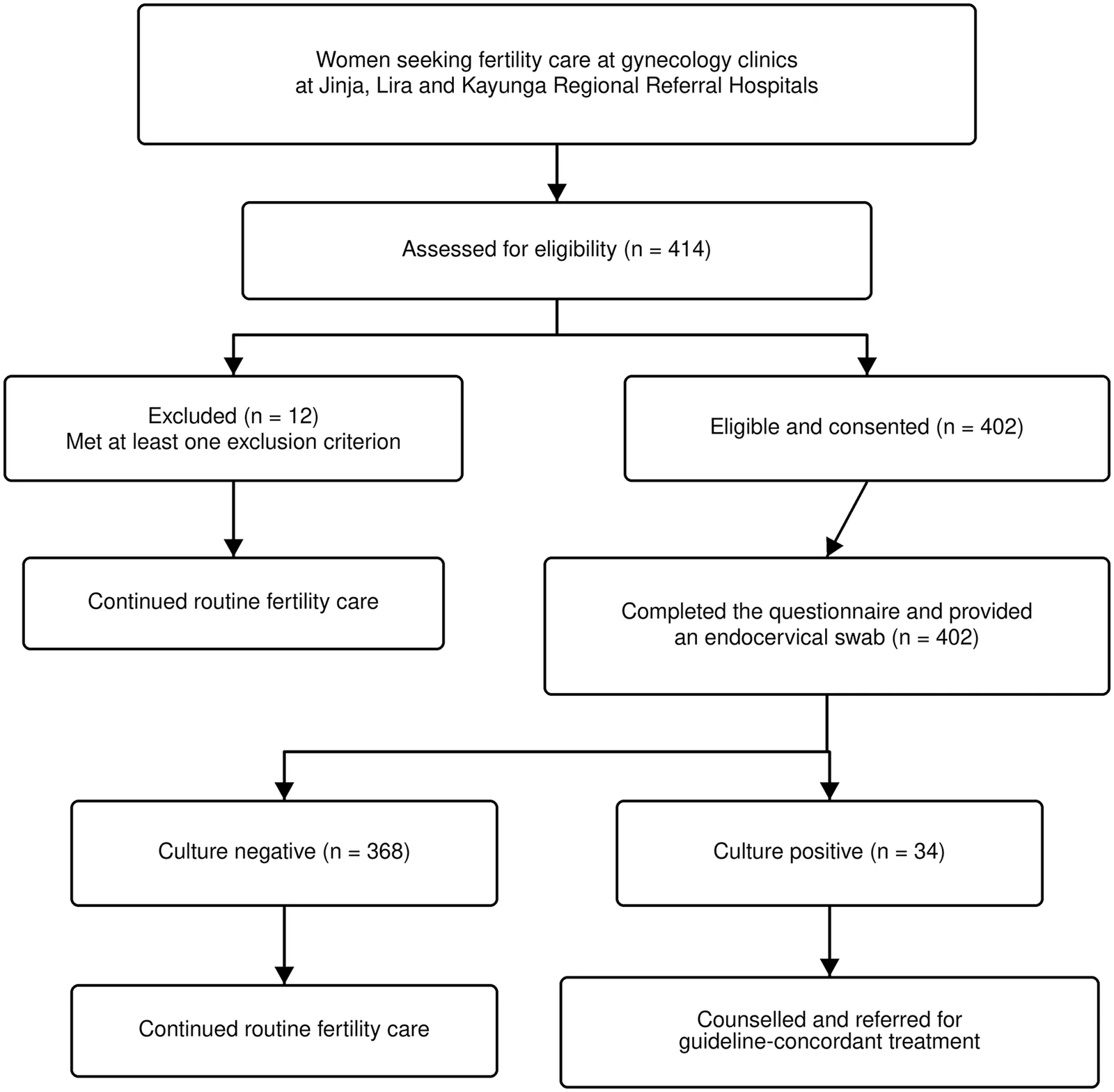
Study flow chart showing participant screening, enrolment, specimen collection, and laboratory outcomes.

A total of 414 women seeking fertility care were assessed for eligibility across the three participating hospitals. Twelve women (2.9%) met at least one exclusion criterion and were not enrolled. The remaining 402 women (97.1%) met the eligibility criteria and all provided written informed consent, giving an eligible-participant response rate of 100%. Each participant completed the questionnaire and provided an endocervical specimen for culture. No enrolled participant was excluded because of incomplete data or a missing laboratory result; therefore, all 402 participants, comprising 134 women from each hospital, were included in the final analysis (Fig 1).

Most participants were aged 30–39 years (252/402, 62.7%), married (264/402, 65.7%), and had primary education (165/402, 41.0%). Informal employment was the most common occupation (177/402, 44.0%), and 178 women (44.3%) reported a monthly household income of less than 200,000 Ugandan shillings. Most participants reported no alcohol use (320/402, 79.6%), first sexual intercourse at 15–17 years (240/402, 59.7%), and no new sexual partner in the preceding 3 months (359/402, 89.3%). A total of 167 women (41.5%) reported three or more lifetime sexual partners, and 76 (18.9%) reported a history of vaginal douching.

A history of abnormal vaginal discharge was reported by 362 women (90.1%). Fourteen participants (3.5%) had a history of ectopic pregnancy. More than half had never used oral contraceptives (234/402, 58.2%), and 221 (55.0%) reported infertility duration of more than 3 years. Most participants were HIV-negative (354/402, 88.1%), 117 (29.1%) reported a history of anemia, and normal body mass index was the most common body mass index category (240/402, 59.7%). Sociodemographic, behavioral, obstetric, gynecologic, and medical characteristics did not differ significantly across the three study sites (Table 1).

**Table 1:** Characteristics of the study participants (N = 402).

Characteristic	Lira RRH n = 134	Jinja RRH n = 134	Kayunga RRH n = 134	Overall N = 402	χ²(df), p-value
	**n (%)**	**n (%)**	**n (%)**	**n (%)**	
**Age group (years)**					Fisher’s exact p = 0.209
<20	2 (1.5)	5 (3.7)	7 (5.2)	14 (3.5)	
20-29	38 (28.4)	31 (23.1)	29 (21.6)	98 (24.4)	
30-39	87 (64.9)	83 (61.9)	82 (61.2)	252 (62.7)	
40+	7 (5.2)	15 (11.2)	16 (11.9)	38 (9.5)	
**Marital status**					χ²(2)=2.05, p=0.358
Not married	47 (35.1)	40 (29.9)	51 (38.1)	138 (34.3)	
Married	87 (64.9)	94 (70.1)	83 (61.9)	264 (65.7)	
**Educational level**					χ²(6)=7.12, p=0.310
None	10 (7.5)	12 (9.0)	10 (7.5)	32 (8.0)	
Primary	57 (42.5)	51 (38.1)	57 (42.5)	165 (41.0)	
Secondary	39 (29.1)	55 (41.0)	48 (35.8)	142 (35.3)	
Tertiary	28 (20.9)	16 (11.9)	19 (14.2)	63 (15.7)	
**Occupation**					χ²(4)=1.30, p=0.861
Informal	60 (44.8)	54 (40.3)	63 (47.0)	177 (44.0)	
Formal	33 (24.6)	35 (26.1)	32 (23.9)	100 (24.9)	
Unemployed	41 (30.6)	45 (33.6)	39 (29.1)	125 (31.1)	
**Monthly household income (USD)**					χ²(4)=2.95, p=0.567
<55USD	60 (44.8)	60 (44.8)	58 (43.3)	178 (44.3)	
55-110USD	37 (27.6)	47 (35.1)	45 (33.6)	129 (32.1)	
>110USD	37 (27.6)	27 (20.1)	31 (23.1)	95 (23.6)	
**Alcohol consumption**					χ²(2)=0.21, p=0.898
No	105 (78.4)	108 (80.6)	107 (79.9)	320 (79.6)	
Yes	29 (21.6)	26 (19.4)	27 (20.1)	82 (20.4)	
**Lifetime number of sexual partners**					χ²(4)=6.28, p=0.179
1 partner	30 (22.4)	44 (32.8)	31 (23.1)	105 (26.1)	
2 partners	45 (33.6)	44 (32.8)	41 (30.6)	130 (32.3)	
≥3 partners	59 (44.0)	46 (34.3)	62 (46.3)	167 (41.5)	
**Age at first sex**					χ²(4)=7.23, p=0.124
<15 years	31 (23.1)	23 (17.2)	36 (26.9)	90 (22.4)	
15-17 years	81 (60.4)	90 (67.2)	69 (51.5)	240 (59.7)	
≥18 years	22 (16.4)	21 (15.7)	29 (21.6)	72 (17.9)	
**New sexual partner in last 3 months**					χ²(2)=0.99, p=0.610
No	117 (87.3)	122 (91.0)	120 (89.6)	359 (89.3)	
Yes	17 (12.7)	12 (9.0)	14 (10.4)	43 (10.7)	
**Previous history of vaginal douching in past 3months**					χ²(2)=4.12, p=0.127
No	102 (76.1)	115 (85.8)	109 (81.3)	326 (81.1)	
Yes	32 (23.9)	19 (14.2)	25 (18.7)	76 (18.9)	
**History of abnormal per vaginal discharge in past 3 months**					χ²(2)=0.89, p=0.641
No	16 (11.9)	12 (9.0)	12 (9.0)	40 (10.0)	
Yes	118 (88.1)	122 (91.0)	122 (91.0)	362 (90.1)	
**History of ectopic pregnancy**					Fisher’s exact p = 0.701
No	129 (96.3)	131 (97.8)	128 (95.5)	388 (96.5)	
Yes	5 (3.7)	3 (2.2)	6 (4.5)	14 (3.5)	
**Oral contraceptive use**					χ²(4)=3.51, p=0.476
Never	77 (57.5)	79 (59.0)	78 (58.2)	234 (58.2)	
Past	36 (26.9)	31 (23.1)	41 (30.6)	108 (26.9)	
Current	21 (15.7)	24 (17.9)	15 (11.2)	60 (14.9)	
**Time since infertility diagnosis**					χ²(4)=7.06, p=0.133
<1 year	13 (9.7)	10 (7.5)	15 (11.2)	38 (9.5)	
1-3 years	45 (33.6)	59 (44.0)	39 (29.1)	143 (35.6)	
>3 years	76 (56.7)	65 (48.5)	80 (59.7)	221 (55.0)	
**HIV status**					χ²(2)=0.57, p=0.753
Negative	120 (89.6)	116 (86.6)	118 (88.1)	354 (88.1)	
Positive	14 (10.4)	18 (13.4)	16 (11.9)	48 (11.9)	
**History of anemia in past 1year**					χ²(2)=1.52, p=0.468
No	96 (71.6)	99 (73.9)	90 (67.2)	285 (70.9)	
Yes	38 (28.4)	35 (26.1)	44 (32.8)	117 (29.1)	
**BMI category (WHO)**					Fisher’s exact p = 0.978
Underweight	8 (6.0)	10 (7.5)	9 (6.7)	27 (6.7)	
Normal	83 (61.9)	77 (57.5)	80 (59.7)	240 (59.7)	
Overweight	38 (28.4)	43 (32.1)	39 (29.1)	120 (29.9)	
Obese	5 (3.7)	4 (3.0)	6 (4.5)	15 (3.7)	

### Prevalence of gonococcal cervicitis

**Fig 2:**
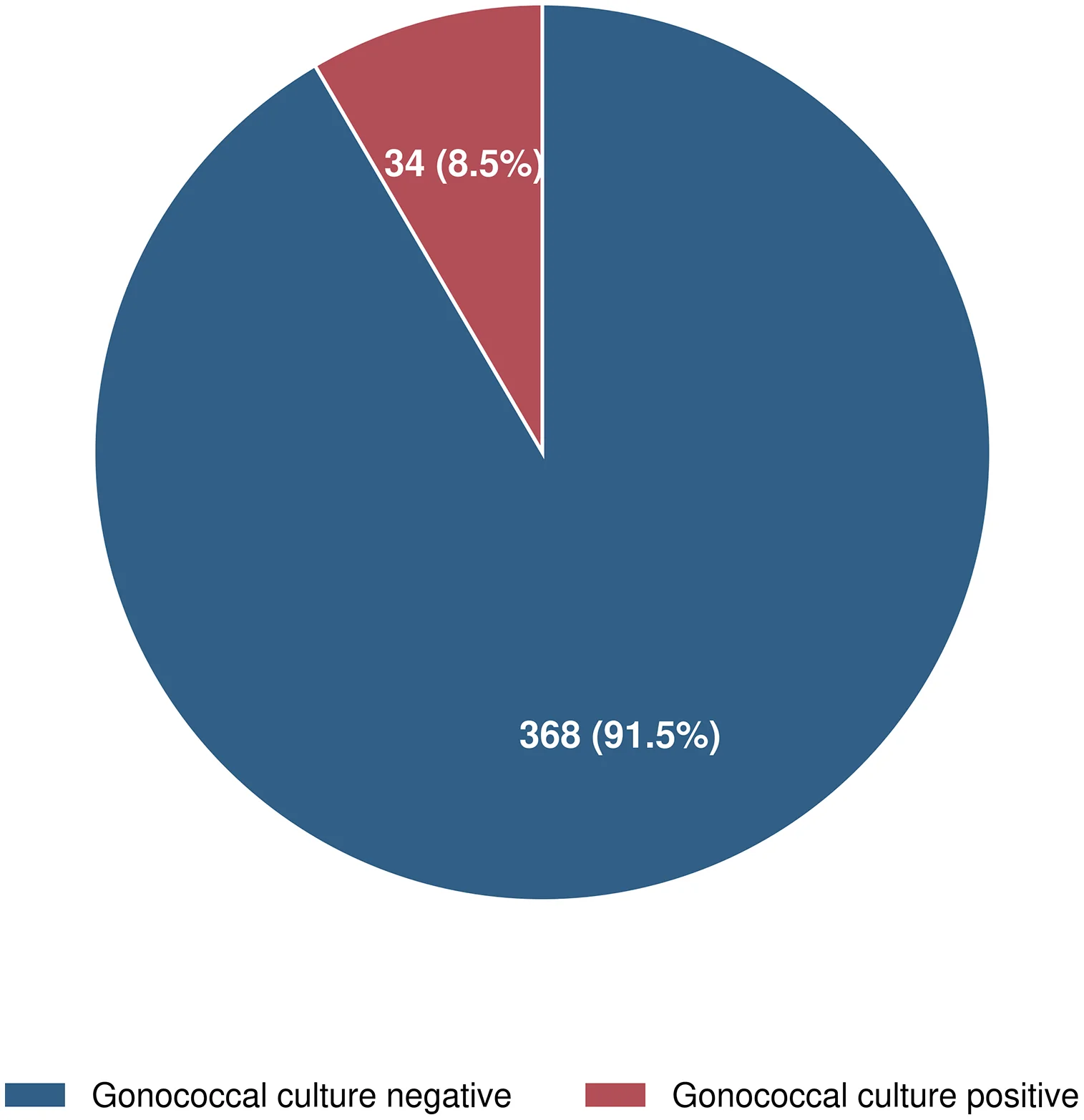
Pie chart showing the overall prevalence of gonococcal cervicitis among women seeking fertility care (N = 402).

Of the 402 women seeking fertility care, 34 had culture-confirmed GC, giving an overall prevalence of 8.5% (95% CI, 6.1%–11.6%). The remaining 368 women (91.5%) were culture-negative (Fig 2).

The prevalence varied modestly by study site. The Jinja Regional Referral Hospital had the highest prevalence at 10.4% (14/134; 95% CI, 6.3–16.9%), while the Lira Regional Referral Hospital and Kayunga Regional Referral Hospital each had a prevalence of 7.5% (10/134; 95% CI, 4.1–13.3%). These differences were not statistically significant (χ² [2] = 1.03, p = 0.598).

### Antimicrobial susceptibility patterns of *Neisseria gonorrhoeae* isolates

Antimicrobial susceptibility results for the 34 culture-positive isolates are shown in Table 2.

**Table 2:** Overall antimicrobial susceptibility patterns of Neisseria gonorrhoeae isolates (n = 34).

Antibiotic	Sensitive n (%)	Intermediate n (%)	Resistant n (%)	Below susceptible only threshold, n (%)
Ceftriaxone	32 (94.1)	NA	1 (2.9)	2(5.9)
Azithromycin	32 (94.1)	NA	0 (0.0)	2(5.9)
Ciprofloxacin	14 (41.2)	0 (0.0)	20 (58.8)	NA
Tetracycline	12 (35.3)	2 (5.9)	20 (58.8)	NA
Penicillin	11 (32.4)	1 (2.9)	22 (64.7)	NA

CLSI provides susceptible-only disk-diffusion thresholds for ceftriaxone (≥35 mm) and azithromycin (≥30 mm). Isolates producing zones below these thresholds were not categorized as intermediate or resistant because MIC confirmation was not performed. NA, not applicable.

Among the 34 isolates tested, 32 (94.1%) met the CLSI susceptible-only disk-diffusion thresholds for ceftriaxone and azithromycin. Two isolates (5.9%) produced zone diameters below the susceptible-only threshold for each agent. Because MIC confirmation was not performed, these findings were reported as reduced zone diameters rather than intermediate or resistant results.

In contrast, resistance was frequent to penicillin, ciprofloxacin, and tetracycline. Resistance was observed in 22 isolates (64.7%) for penicillin and 20 isolates (58.8%) each for ciprofloxacin and tetracycline. Susceptibility was observed in 14 isolates (41.2%) for ciprofloxacin, 12 (35.3%) for tetracycline, and 11 (32.4%) for penicillin. Two tetracycline isolates (5.9%) and one penicillin isolate (2.9%) had intermediate results; no intermediate result was observed for ciprofloxacin.

### Factors associated with gonococcal cervicitis

#### Bivariable analysis.

In the bivariable logistic regression, several variables met the prespecified screening threshold for inclusion in the multivariable model. These included age group, monthly household income, history of ectopic pregnancy, duration of infertility, body mass index category, number of lifetime sexual partners, history of vaginal douching, oral contraceptive use, HIV status, and reporting a new sexual partner in the previous three months (Table 3).

**Table 3:** Bivariable logistic regression of factors associated with gonococcal cervicitis (selected variables shown).

Characteristic	Gonococcal culture negative n (%)	Gonococcal culture positive n (%)	COR	95% CI	p-value
**Age group (years)**					
20-29	91 (24.7)	7 (20.6)	1.00	ref	
<20	11 (3.0)	3 (8.8)	3.55	0.80-15.76	0.096
30-39	233 (63.3)	19 (55.9)	1.06	0.43-2.61	0.899
≥40	33 (9.0)	5 (14.7)	1.97	0.58-6.65	0.275
**Monthly household income (USD)**					
>110USD	82 (22.3)	13 (38.2)	1.00	ref	
<55USD	167 (45.4)	11 (32.4)	0.42	0.18-0.97	0.042
55-110USD	119 (32.3)	10 (29.4)	0.53	0.22-1.27	0.154
**Lifetime number of sexual partners**					
1 partner	101 (27.4)	4 (11.8)	1.00	ref	
2 partners	122 (33.2)	8 (23.5)	2.53	0.67-9.61	0.173
≥3 partners	145 (39.4)	22 (64.7)	5.16	1.50-17.72	0.009
**History of vaginal douching in past 3 months**					
No	308 (83.7)	18 (52.9)	1.00	ref	
Yes	60 (16.3)	16 (47.1)	4.56	2.20-9.46	<0.001
**History of ectopic pregnancy**					
No	358 (97.3)	30 (88.2)	1.00	ref	
Yes	10 (2.7)	4 (11.8)	4.77	1.41-16.16	0.012
**Oral contraceptive use**					
Never	223 (60.6)	11 (32.4)	1.00	ref	
Past	99 (26.9)	9 (26.5)	1.84	0.74-4.59	0.190
Current	46 (12.5)	14 (41.2)	6.17	2.63-14.47	<0.001
**Time since Infertility diagnosis**					
<1 year	35 (9.5)	3 (8.8)	1.00	ref	
1-3 years	139 (37.8)	4 (11.8)	0.34	0.07-1.57	0.166
>3 years	194 (52.7)	27 (79.4)	1.62	0.47-5.65	0.446
**HIV status**					
Negative	332 (90.2)	22 (64.7)	1.00	ref	
Positive	36 (9.8)	12 (35.3)	5.03	2.30-11.02	<0.001
**History of anemia in past 1 year**					
No	257 (69.8)	28 (82.4)	1.00	ref	
Yes	111 (30.2)	6 (17.6)	0.50	0.20-1.23	0.131
**Body mass index category (WHO)**					
Normal	223 (60.6)	17 (50.0)	1.00	ref	
Underweight	26 (7.1)	1 (2.9)	0.50	0.06-3.96	0.515
Overweight	107 (29.1)	13 (38.2)	1.59	0.75-3.40	0.229
Obese	12 (3.3)	3 (8.8)	3.28	0.84-12.77	0.087
**New sexual partner in last 3 months**					
No	334 (90.8)	25 (73.5)	1.00	ref	
Yes	34 (9.2)	9 (26.5)	3.54	1.53-8.20	0.003

#### Multivariable analysis.

In the multivariable logistic regression, five variables remained independently associated with GC (Table 4). Women reporting three or more lifetime sexual partners had significantly higher odds of gonococcal cervicitis than women reporting one partner (adjusted odds ratio [AOR], 5.25; 95% CI, 1.65–16.72; p = 0.005). A history of vaginal douching remained strongly associated with infection (AOR, 5.59; 95% CI, 1.83–17.03; p = 0.002). Current oral contraceptive use was independently associated with gonococcal cervicitis (AOR, 8.32; 95% CI, 2.94–23.54; p < 0.001), whereas past oral contraceptive use was not (AOR, 1.44; 95% CI, 0.43–4.87; p = 0.555). HIV-positive women had higher odds of gonococcal cervicitis than HIV-negative women (AOR, 5.00; 95% CI, 1.82–13.76; p = 0.002). Reporting a new sexual partner in the preceding 3 months was independently associated with infection (AOR, 7.92; 95% CI, 2.68–23.41; p < 0.001).

**Table 4:** Multivariable logistic regression of factors associated with gonococcal cervicitis.

Characteristic	COR (95% CI)	p-value	AOR (95% CI)	p-value
**Age group (years)**				
20-29	1.00		1.00	
<20	3.55 (0.80-15.76)	0.096	3.81 (0.58-25.12)	0.164
30-39	1.06 (0.43-2.61)	0.899	0.72 (0.23-2.25)	0.570
≥40	1.97 (0.58-6.65)	0.275	2.07 (0.43-10.02)	0.368
**Monthly household income (USD)**				
>110USD	1.00		1.00	
<55USD	0.42 (0.18-0.97)	0.042	0.46 (0.16-1.34)	0.156
55-110USD	0.53 (0.22-1.27)	0.154	0.62 (0.19-1.99)	0.420
**Lifetime number of sexual partners**				
1 partner	1.00		1.00	
2 partners	2.53 (0.67-9.61)	0.173	1.24 (0.30-5.15)	0.771
≥3 partners	5.16 (1.50-17.72)	0.009	5.25 (1.65-16.72)	0.005
**History of vaginal douching in past 3months**				
No	1.00		1.00	
Yes	4.56 (2.20-9.46)	<0.001	5.59 (1.83-17.03)	0.002
**History of ectopic pregnancy**				
No	1.00		1.00	
Yes	4.77 (1.41-16.16)	0.012	4.07 (0.87-19.07)	0.075
**Oral contraceptive use**				
Never	1.00		1.00	
Past	1.84 (0.74-4.59)	0.190	1.44 (0.43-4.87)	0.555
Current	6.17 (2.63-14.47)	<0.001	8.32 (2.94-23.54)	<0.001
**Time since Infertility diagnosis**				
<1 year	1.00		1.00	
1-3 years	0.34 (0.07-1.57)	0.166	0.44 (0.07-2.65)	0.369
>3 years	1.62 (0.47-5.65)	0.446	2.29 (0.51-10.31)	0.280
**HIV status**				
Negative	1.00		1.00	
Positive	5.03 (2.30-11.02)	<0.001	5.00 (1.82-13.76)	0.002
**Body mass index category (WHO)**				
Normal	1.00		1.00	
Underweight	0.50 (0.06-3.96)	0.515	0.30 (0.04-2.48)	0.262
Overweight	1.59 (0.75-3.40)	0.229	1.58 (0.61-4.09)	0.341
Obese	3.28 (0.84-12.77)	0.087	1.67 (0.10-28.10)	0.721
**New sexual partner in last 3 months**				
No	1.00		1.00	
Yes	3.54 (1.53-8.20)	0.003	7.92 (2.68-23.41)	<0.001

Age group, monthly household income, history of ectopic pregnancy, duration of infertility, and body mass index category did not retain statistical significance after adjustment. The final multivariable logistic regression model showed no evidence of lack of fit on the Hosmer–Lemeshow goodness-of-fit test (χ² = 3.16, df = 8, p = 0.924). Neither of the prespecified multiplicative interactions was supported: HIV status × new sexual partner (LR χ²(1) = 0.17, p = 0.680) or vaginal douching × abnormal vaginal discharge (LR χ²(1) = 0.30, p = 0.584). The interaction terms were therefore not retained in the final model. Given the small number of culture-positive outcomes and the limited numbers in some joint-exposure categories, these analyses were considered exploratory.

## Discussion

This Multicenter cross-sectional study found culture-confirmed gonococcal cervicitis in 34 of 402 women seeking fertility care at three regional referral hospitals in Uganda, corresponding to a prevalence of 8.5%. Among the 34 isolates, 32 (94.1%) met the susceptible-only disk-diffusion thresholds applied for ceftriaxone and azithromycin, while two isolates (5.9%) produced inhibition zones below the susceptible threshold for each agent. Resistance was common to penicillin (64.7%), ciprofloxacin (58.8%) and tetracycline (58.8%). After adjustment, gonococcal cervicitis was associated with three or more lifetime sexual partners, a new sexual partner during the preceding three months, vaginal douching, current oral contraceptive use and HIV seropositivity.

The prevalence observed in this study was above the pooled African estimate of 5.0% reported among infertile populations, although it remained within the meta-analysis confidence interval of 1.9%–9.3% [11]. It was substantially higher than the prevalence reported among women preparing for pregnancy in Chongqing and in a population-based study in Shenzhen, where prevalence estimates were 0.06% and 0.17%, respectively [14,15]. Conversely, the present estimate was lower than the 23.6% reported among patients attending outpatient clinics at St. Mary’s Hospital Lacor in northern Uganda [17]. These estimates should not be interpreted as direct geographical comparisons because the studies differed considerably in participant selection, symptom profile, healthcare setting and diagnostic method. Nevertheless, they suggest that women attending fertility services may carry an appreciable burden of gonococcal infection even when they are not seeking care primarily because of symptoms.

Within East Africa, the present prevalence was close to the 10.0% reported among patients attending private clinics in Mekelle and the 9.8% reported among clinically suspected patients in Jimma, Ethiopia [18,20]. It was somewhat higher than the 4.9% reported among symptomatic patients in Lyantonde, Uganda, and the 6.3% detected by polymerase chain reaction among symptomatic female sex workers attending an STI clinic in Nairobi [16,19]. Variation between these estimates may reflect differences in recruitment criteria, symptom profiles, sexual networks, previous antibiotic exposure and diagnostic sensitivity. The studies also involved markedly different populations, ranging from women seeking fertility care to symptomatic clinic attendees and female sex workers. Their prevalence estimates should therefore be interpreted within their respective clinical and epidemiological contexts.

The fertility-care setting is particularly important when interpreting the findings. Untreated *Neisseria gonorrhoeae* infection can ascend to the upper genital tract, cause pelvic inflammatory disease and contribute to tubal damage, ectopic pregnancy and infertility [2,4,5]. However, the present study detected current infection after infertility had already been diagnosed. Its cross-sectional design cannot establish that the infections detected during enrolment preceded or caused infertility. A positive culture could represent persistent infection, reinfection or a more recent exposure occurring after infertility had developed. The findings therefore demonstrate coexistence of gonococcal infection and infertility but should not be interpreted as estimating the proportion of infertility directly attributable to gonorrhoea.

The use of culture may also have influenced the measured prevalence. Culture was necessary because viable isolates were required for phenotypic antimicrobial susceptibility testing. However, culture is generally less sensitive than nucleic acid amplification testing and depends on organism viability, appropriate specimen collection, suitable transport conditions and timely laboratory processing. NAATs generally offer greater sensitivity, particularly when the bacterial load is low or infection is asymptomatic [36]. Therefore, the 8.5% estimate represents culture-confirmed infection and may not capture every current infection in this population. Future studies combining NAAT-based detection with culture of positive specimens would provide a more complete estimate while preserving viable isolates for antimicrobial-resistance surveillance.

The high resistance to penicillin, ciprofloxacin and tetracycline is consistent with previous Ugandan evidence. In Kampala, Mabonga et al found that all viable gonococcal isolates were resistant to ciprofloxacin and tetracycline by disk diffusion [32]. Uganda’s Enhanced Gonococcal Antimicrobial Surveillance Programme subsequently documented greater than 90% resistance to ciprofloxacin, tetracycline and penicillin among men presenting with urethral discharge syndrome [37]. Although these studies involved different patient populations and laboratory procedures, their findings and those of the present study consistently demonstrate the limited value of these older agents for empirical gonorrhoea treatment. This pattern also agrees with systematic-review and global-surveillance evidence showing widespread gonococcal resistance to penicillin, tetracycline and fluoroquinolones [24,30,31].

Most isolates in the present study met the susceptible-only disk-diffusion thresholds applied for ceftriaxone and azithromycin. However, two isolates produced inhibition zones below the susceptible threshold for each agent. These findings should be reported as reduced zone diameters rather than confirmed intermediate or resistant results because corresponding intermediate and resistant disk-diffusion categories were not applied, and MIC confirmation was not performed [38]. Reduced inhibition zones alone do not establish clinical resistance or predict treatment failure.

The need for cautious interpretation is supported by previous Ugandan findings. Mabonga et al. reported that some isolates appeared to have reduced ceftriaxone and cefixime susceptibility by disk diffusion but remained susceptible when tested by reference agar dilution [32]. Workneh et al also reported occasional disagreement between disk-diffusion and E test results in Uganda’s gonococcal surveillance Programme [37]. Therefore, the present ceftriaxone and azithromycin findings should not be described as definitive evidence of preserved susceptibility or emerging resistance. Instead, they indicate that isolates producing reduced zone diameters require confirmation using a validated MIC method. This interpretation is especially important because ceftriaxone remains central to recommended gonorrhoea treatment, while resistance or reduced susceptibility to extended-spectrum cephalosporins has been reported internationally [27,30,31].

The substantial resistance to older agents also has wider public-health implications. Treatment options for gonorrhoea have progressively narrowed as *N. gonorrhoeae* has developed resistance to successive antimicrobial classes [29,39]. Continued, quality-assured surveillance is therefore essential for detecting changes in susceptibility and informing national treatment recommendations. Surveillance is particularly important where syndromic management remains common because women with asymptomatic cervical infection may remain untreated, while patients without organism-specific confirmation may receive antibiotics unnecessarily. Expanding access to reliable diagnosis, culture and MIC confirmation would strengthen antimicrobial stewardship and provide more dependable evidence for Ugandan treatment guidelines [32,37]. Women reporting three or more lifetime sexual partners and those reporting a new sexual partner during the preceding three months had higher odds of gonococcal cervicitis. These findings are epidemiologically plausible because a greater number of partners increases the opportunity for exposure, while a recent partnership may introduce contact with a different sexual network. Similar associations with multiple sexual partners have been reported in Mekelle and among female sex workers in Nairobi [16,18]. These characteristics should be understood as indicators of exposure opportunity rather than behaviors that inevitably lead to infection. Their importance supports confidential and non-judgmental assessment of recent partnerships, condom use and STI-prevention needs within fertility services.

HIV seropositivity was also independently associated with gonococcal cervicitis. A systematic review and meta-analysis from sub-Saharan Africa estimated a pooled gonorrhoea prevalence of 3.5% among women living with HIV and found curable STIs to be more prevalent among women with HIV than among women without HIV [40]. HIV and gonorrhoea may cluster within overlapping sexual networks. In addition, gonococcal infection can promote genital inflammation and epithelial-barrier disruption, which may facilitate HIV transmission and increase genital HIV shedding in people with coinfection [41]. However, the present cross-sectional data cannot determine the direction of the association or establish that HIV infection itself increased susceptibility to gonorrhoea. The finding nevertheless supports closer integration of laboratory-based STI assessment with HIV and fertility care.

Vaginal douching remained independently associated with gonococcal cervicitis. Douching may alter the cervicovaginal environment and contribute to the loss of stable, Lactobacillus-dominated microbial communities [21–23]. Longitudinal evidence has shown that regular douching is associated with disruption of the vaginal flora and a greater risk of bacterial vaginosis [42]. However, direct epidemiological evidence linking douching to gonococcal infection is inconsistent. A prospective study of women at high risk found no statistically significant association between douching and incident gonococcal or chlamydial infection [43]. Reverse causation is also possible in the present study because some women may have douched in response to discharge, odor or other genital symptoms. The association should therefore be treated as hypothesis-generating rather than proof that douching directly causes gonococcal infection. Nevertheless, respectful counselling about avoiding potentially disruptive intravaginal practices may be appropriate within fertility and reproductive-health services.

The association with current oral contraceptive use also requires cautious interpretation. A systematic review and meta-analysis found a negative but statistically non-significant association between hormonal contraceptive use and gonorrhoea, meaning that the broader evidence does not establish hormonal contraception as a cause of gonococcal infection [44]. In the present study, oral contraceptive use may have been related to unmeasured differences in condom use, partnership patterns, healthcare contact or other behavioural characteristics. Residual confounding and chance also remain possible, particularly given the modest number of culture-positive participants. This finding should therefore not be used to discourage oral contraceptive use. Instead, contraceptive counselling should emphasize that oral contraceptives prevent pregnancy but do not protect against sexually transmitted infections.

Broader sociodemographic characteristics did not remain independently associated with gonococcal cervicitis after adjustment. This may indicate that recent sexual exposures and clinical factors were more informative than age, education, marital status or income in this fertility-care population. However, non-significant associations should not be interpreted as evidence that these factors have no relationship with infection. Only 34 participants had positive cultures, which limited statistical precision and may have reduced the study’s ability to identify weaker associations. The adjusted findings should therefore be interpreted cautiously and confirmed in larger prospective studies.

Overall, the study demonstrates that gonococcal cervicitis and resistance to older antimicrobial agents remain clinically relevant among women seeking fertility care in Uganda. The findings support closer integration of fertility, STI and HIV services, including confidential sexual-risk assessment, laboratory testing where feasible, partner management and antimicrobial-resistance surveillance. Nevertheless, a single cross-sectional study is insufficient to recommend universal screening in all fertility clinics. Larger studies using NAAT alongside culture, validated MIC testing and prospective follow-up are needed to determine the burden of undiagnosed infection, clarify temporal relationships with infertility and evaluate the feasibility and cost-effectiveness of laboratory-based screening within fertility services.

### Study strengths and limitations

This Multicenter study included three tertiary hospitals from geographically distinct regions of Uganda, strengthening its relevance to public fertility-care services. It assessed prevalence, antimicrobial susceptibility and associated factors within the same population, allowing the clinical, laboratory and epidemiological findings to be interpreted together. Culture-based diagnosis also provided viable isolates for phenotypic antimicrobial susceptibility testing rather than relying on symptoms or syndromic diagnosis alone.

Several limitations should be considered. The cross-sectional design prevents determination of whether the identified behavioural and clinical factors preceded infection or whether the detected infections contributed to infertility. Because recruitment was limited to women attending tertiary fertility clinics, the findings may not be generalizable to women who do not seek fertility care or who attend lower-level facilities. Culture is less sensitive than nucleic acid amplification testing, particularly when bacterial load is low or specimen transport and processing are suboptimal; therefore, the reported prevalence may underestimate the true burden of infection. Behavioural and reproductive information was self-reported and remains susceptible to recall and social-desirability bias despite the use of private interviews and neutral questioning.

The sample-size calculation did not account for possible clustering within hospitals. Although the achieved sample exceeded the individually calculated minimum and prevalence did not differ significantly across hospitals, residual hospital-level correlation cannot be excluded. In addition, only 34 participants had positive cultures, limiting the precision of the antimicrobial-susceptibility estimates and multivariable associations.

Antimicrobial susceptibility was assessed by disk diffusion without MIC confirmation. Consequently, isolates producing ceftriaxone or azithromycin inhibition zones below the susceptible-only thresholds could not be classified as confirmed resistant. Run-level quality-control measurements and documentation of repeat testing were also unavailable in the study dataset; therefore, quality-control discordance could not be assessed retrospectively. The small number of culture-positive outcomes limited the power to detect interaction; therefore, the absence of statistically significant interaction terms should not be interpreted as evidence that effect modification does not exist. Only 34 culture-positive outcomes were observed. Consequently, multivariable estimates remained vulnerable to sparse-data bias, limited precision, and model instability even after restricting the number of predictors and using penalized regression. The associated factors should therefore be interpreted as exploratory and require confirmation in larger studies.

### Implications and future research

The coexistence of gonococcal infection, HIV and resistance to older antimicrobial agents highlights an opportunity to strengthen integration of fertility, STI and HIV services. Where feasible, fertility care could incorporate laboratory-based STI assessment, risk-reduction counselling, partner management and antimicrobial-resistance surveillance. However, the findings from this single cross-sectional study are insufficient to recommend universal routine screening.

Larger prospective studies should evaluate the feasibility and cost-effectiveness of gonococcal screening in fertility clinics, include sensitive molecular testing for *N. gonorrhoeae* and coexisting infections such as *Chlamydia trachomatis*, and investigate reproductive outcomes following diagnosis and treatment. Future antimicrobial-resistance studies should use validated MIC methods to confirm reduced ceftriaxone and azithromycin susceptibility.

## Conclusions and recommendations

Gonococcal cervicitis was common among women seeking fertility care at the participating Ugandan hospitals. Resistance to penicillin, ciprofloxacin and tetracycline was substantial, while most isolates met the susceptible-only thresholds for ceftriaxone and azithromycin. Isolates with reduced ceftriaxone or azithromycin inhibition zones require MIC confirmation before resistance can be established.

Infection was associated with HIV seropositivity, multiple lifetime sexual partners, a recent new sexual partner, vaginal douching and current oral contraceptive use. These findings support integrating laboratory-based STI assessment, partner management, risk-reduction counselling and antimicrobial-resistance surveillance into fertility and HIV services. Larger prospective studies using sensitive molecular diagnostics and validated MIC methods are needed before routine gonococcal screening in fertility clinics can be recommended.

## Supporting information

S1 Data Fully anonymized participant-level dataset supporting the findings of this study. (XLSX)

S1 Text Structured, pretested, interviewer-administered questionnaire used to collect participants’ sociodemographic characteristics, sexual and behavioural exposures, reproductive and gynecological history, clinical factors, and relevant medical information. (DOCX)
