# Supplementary material for: Gonococcal cervicitis among women seeking fertility care in Uganda: Prevalence, antimicrobial susceptibility patterns, and associated factors in a multicenter cross-sectional study
Source: PLOS Glob Public Health. 2026 Sep 8;6(9):e0006796. doi: 10.1371/journal.pgph.0006796 (PMC13552739; doi:10.1371/journal.pgph.0006796)
Supplement: Supplementary file 1 [file pgph.0006796.s002.docx]

**APPENDIX I: INVESTIGATOR ADMINISTERED QUESTIONNAIRE-ENGLISH**

**Study:**" **PREVALENCE, SUSCEPTIBILITY PATTERNS, AND FACTORS ASSOCIATED WITH GONOCOCCAL CERVICITIS AMONG WOMEN SEEKING FERTILITY CARE AT TERTIARY HOSPITALS IN UGANDA.**

**Date of Completion:** _________________________

**Participant ID:**

**Section A: Socio-Demographic Characteristics**

Age: ____ years

Marital status:
 - [ ] Single
 - [ ] Married
 - [ ] Divorced
 - [ ] Widowed
 - [ ] Polygamous

Educational level:
 - [ ] No formal education
 - [ ] Primary
 - [ ] Secondary
 - [ ] Tertiary

Occupation: ____________________

Household income per month: ____________________

Alcohol consumption:
 - [ ] Yes
 - [ ] No

Smoking:
 - [ ] Yes
 - [ ] No

**Section B: Obstetric and Medical Factors**

Number of lifetime sexual partners: _____

Age at first sexual intercourse: _____

History of abnormal vaginal discharge:
 - [ ] Yes
 - [ ] No

History of douching practices:
 - [ ] Yes
 - [ ] No

History of ectopic pregnancy:
 - [ ] Yes
 - [ ] No

Use of oral contraceptives:
 - [ ] currently using
 - [ ] Used in the past
 - [ ] Never used

Duration of infertility:
 - [ ] Less than 1 year
 - [ ] 1–3 years
 - [ ] More than 3 years

Sexual behavior (multiple partners, transactional sex, new partner in last 3 months, etc.

**SECTION C: Medical History**

HIV status:
 - [ ] Positive
 - [ ] Negative
 - [ ] Not sure

History of anemia diagnosis:
 - [ ] Yes
 - [ ] No

Measured BMI: _____ kg/m²

**Section D: Dependent Variable (Gonococcal cervicitis)**

1. Cervical swab sample taken:
 - [ ] Yes
 - [ ] No

2. Gonococcal culture result:
 - [ ] Positive
 - [ ] Negative

3. Antibiotic susceptibility profile (tick all that apply):

- Ceftriaxone: [ ] Sensitive [ ] Resistant

- Azithromycin: [ ] Sensitive [ ] Resistant

- Ciprofloxacin: [ ] Sensitive [ ] Resistant

- Tetracycline: [ ] Sensitive [ ] Resistant

- Penicillin: [ ] Sensitive [ ] Resistant

- Others: _____________________
